# Extended postpartum intimate partner violence and its associated factors: community-based cross-sectional study design

**DOI:** 10.1186/s12905-023-02649-w

**Published:** 2023-09-20

**Authors:** Lema Fikadu Wedajo, Abera Mersha Mamo, Solomon Seyife Alemu, Bezawit Afework Mesfin

**Affiliations:** 1 Department of Midwifery, College of medical and Health Sciences, Mettu University, Metu, Ethiopia; 2 Departments of Nursing, College of Medicine and Health Sciences, Arba Minch University, Arba Minch, Ethiopia; 3 Departments of Midwifery, College of Medicine and Health Sciences, Arba Minch University, Arba Minch, Ethiopia

**Keywords:** Community-based, Extended postpartum, Intimate partner, Violence

## Abstract

**Background:**

Intimate partner violence is a global problem that threatens mothers. It has multidimensional consequences but has not gained attention from scholars after childbirth.

**Objective:**

To assess the prevalence of extended postpartum intimate partner violence and its associated factors.

**Method:**

A community-based cross-sectional study design was employed among 570 postpartum mothers in Arba Minch Town, Southern Ethiopia, from May 21st to June 21st, 2022. A pretested, face-to-face interviewer-administered structured questionnaire was used. Bivariable and multivariable logistic regression analyses were used. The level of statistical significance was declared at P < 0.05 with a 95% CI.

**Results:**

Overall, the prevalence of extended postpartum intimate partner violence was 45% (95% CI: 40.89, 49.20). Participants whose husband has no formal education (AOR = 3.62; 95%CI: 1.32, 9.90) and only secondary education (AOR = 2.96; 95%CI: 1.56, 5.48), husband alcohol consumption (AOR = 1.73; 95%CI: 1.06, 2.80), husband dominance in decision-making (AOR = 1.94; 95%CI: 1.13, 3.33), husband disappointment in the gender of the baby (AOR = 2.13; 95%CI: 1.28, 3.56), previous history of intimate partner violence (AOR = 5.71; 95%CI: 3.59, 9.07), and low social support (AOR = 4.37; 95%CI: 2.53, 7.55) were significantly associated factors.

**Conclusions and recommendations:**

The prevalence of extended postpartum intimate partner violence was found to be high. Thus, increasing awareness of husbands with no formal education and having lower academic achievement, incorporating maternal social support assessment into maternity and child health care; teaching on alcohol reduction behavior and gender roles; and screening of mothers during the prenatal period should be given.

**Supplementary Information:**

The online version contains supplementary material available at 10.1186/s12905-023-02649-w.

## Introduction

Intimate partner violence is a pervasive social and public health issue that endangers women’s physical, emotional, sexual, and reproductive health and occurs among people in a romantic relationship [1, 2]. It can be explained in the form of physical, sexual, psychological, control of behavior, and economic violence [3–5].

Physical intimate partner violence can be defined as the intentional use of force to cause harm, disability, and death [3]. However, sexual intimate partner violence is an act of sexual attempt by an intimate partner, like forced sex, practicing sex due to fear of the husband, or doing something sexual without her interest [3]. In addition, control of behavior can be explained when an intimate partner suspects his spouse of being unfaithful to him, gets angry while she speaks with another man, insists on knowing where she goes every time, and prevents her from visiting family of birth and friends [4].

Furthermore, psychological violence can be defined as when the husband insults, belittles, humiliates, and threatens or tries to threaten the person she loves or cares for [4]. Whereas economic violence is the condition in which the husband takes her earnings or prevents her from having money while he has it for household or other expenses [5, 6].

During the postpartum period, there are significant hormonal and physiological changes. Furthermore, it is a period of significant psychosocial change as she adjusted to motherhood, reestablished relationships, and worked to meet the physical and emotional demands of her infant and family members. As a result, postpartum mothers quality of life may be impacted, potentially leading to conflicts between intimate partner that may cause intimate partner violence during the postpartum period [7–9].

Globally, 27% of ever-married women aged 15 to 49 have experienced intimate partner violence in their lifetime [1]. The survey conducted in the United States of America showed that 33.8% of mothers were exposed to intimate partner violence, of which 47.2% were newly emerging types of violence after childbirth [10]. Furthermore, studies conducted in Australia, Brazil, and Iran showed that postpartum mothers suffered from the highest magnitude of intimate partner violence after childbirth, which ranged from 17.4 to 58% [11–13]. Similarly, a pocket of studies conducted in Africa showed the largest magnitude of intimate partner violence after childbirth, which ranged from 8.1 to 46% [14, 15].

Despite the fact that it has multidimensional effects on the world as a whole, little attention was given to intimate partner violence during the postpartum period in both high- and low-income countries. Mothers exposed to intimate partner violence were at a higher risk of common mental health problems like anxiety and suicidal ideation [16, 17]. Studies showed that mothers exposed to intimate partner violence were at higher risk of sexually transmitted infections like Human Immune Virus (HIV) and cervical cancer, which might affect the value of maternity as it leads to problems in infant nurturing [18–20].

Infants of mothers exposed to intimate partner violence have a lower chance of continuing exclusive breast feeding and getting a minimum acceptable diet during infancy, and it might affect the immunization status of infants as per recommendations. Intimate partner violence also increases childhood morbidities like diarrhea, acute respiratory tract infections, and fever during infancy, which affect the future generation that will inherit this world [20–24].

Different international organizations like the CDC and WHO have proposed strategies to overcome and eradicate intimate partner violence [25, 26], but currently, it is at the climax stage and affects both the health and economies of the world. Even though it is a strong political agenda and human rights issue worldwide, the Ethiopian government ministry of health (MOH) has not incorporated it into the program of health sector transformation plan Two [27].

The postpartum period is critical period in both the mother’s and the infants live. In addition, new generations emerge from partners with this problem become the perpetrators of the problem when their term reaches as they inherit from the families of birth. This may affect the world agenda, i.e., Sustainable Development Goal 5.2, which focuses on the eradication of any form of gender-based violence by 2030. Thus, the only way to eradicate intimate partners violence is to enrich the new generations with family love.

It was not researched as much as should be, viewing the seriousness of the problem among extended postpartum mothers. Therefore, this study aimed to assess the prevalence and associated factors of extended postpartum intimate partner violence in Arba Minch town, 2022. This helps to identify the existing gap and improve the utilization of screening for postpartum intimate partner violence in this population category based on identified factors.

## Methods and materials

### Study design, area, and period

A community-based cross-sectional study design was employed in Arba Minch town. The town has 12 kebele, the smallest administrative unit of the town. Arba Minch town is the administrative city of the Gamo zone and the homeland of Nech-Sar Park. This town was 505 km south of the capital city of the country, Addis Ababa. It is found in the rift valley above the sea level of 1285 m. Arba Minch town has a total population of 112,724 in 24,090 households. The total number of reproductive-age women in this town is 56,137, and the expected delivery is 4,272. The study was conducted on postpartum mothers who were in the first year after childbirth from May 21st to June 21st, 2022.

### Inclusion criteria and exclusion criteria

All postpartum mothers who were in their first year after birth during the data collection period were included in this study. Those mothers who were critically ill during the data collection period and whose husbands were diagnosed with known mental health problems were excluded from the study.

### Sample size and sampling technique

The sample size for this study was determined by using the single population proportion formula, taking the proportion of postpartum intimate partner violence as 31.4% [28], which was conducted in central Ethiopia using a 95% confidence interval and a 4% margin of error (d) as follows: n$=\frac{(\text{z}{\upalpha }/2)2\left(\text{p}\text{q}\right)}{\text{d}2 }$, n =$\frac{ \left(1.96\right)2\left(0.314\left)\right(0.686\right)}{\left(0.04\right)2}$; n = 518. Finally, by adding a 10% nonresponse rate, the final sample size of 570 was taken.

A simple random sampling technique was used to select the study populations. Initially, the total numbers of postpartum mothers who were in the first year (from May 21st, 2021, to May 21st, 2022) was obtained from each kebele. The total delivery of Arba Minch Town from May 21st, 2021, to May 21st, 2022, was 3963. Then, to obtain the total sample size, proportional allocation of postpartum mothers from each kebele was done, and then the total delivery of each kebele was converted to a table of random numbers. Finally, by a computer-generated simple random sampling method, proportionally allocated mothers were selected from each kebele, and the selected mothers’ houses were coded with the help of each kebele health extension worker before starting actual data collection.

### Study variables

The dependent variable for this study was the prevalence of extended postpartum intimate partner violence. The independent variables were socio-demographic variables (age of both couple, educational status of husband and wife, family size, occupation of wife, type of marriage ceremony, living apart from husband, choice of husband by women and dowry payment), couples behavioral and substance related factors (alcohol, Khat, smoking and physical fights), reproductive history related factors (husband disappointment with a gender of current baby, husband index of pregnancy during current baby, age at marriage, length of marriage, miscarriage and still birth history), and maternal personnel and social related factors (maternal depression, history intimate partner violence during and/or before index of pregnancy, maternal social support and husband dominance in decision-making authority) was assessed.

### Operational definitions, definition of terms and measurements

Prevalence of extended postpartum intimate partner violence: Defined according to this study as if the mother faced at least one type of intimate partner violence during her first year after the birth of a viable baby. It was coded as ‘0’if she was not the victim and ‘1’if she was the victim of the problem during her first year after childbirth [6].

Physical violence was assessed by six questions; if she responded yes to at least one question, she was the victim of physical intimate partners violence; and psychological violence was assessed by four questions, and the mothers experienced at least one type of question suffering from it [5]. In addition, control of behavior was assessed by five questions; if she responded yes to one question, she was the victim of control of behavior; and economic violence was assessed by two questions; if the response to at least one question was yes, she was the victim of it [5]. Furthermore, sexual violence was assessed by three questions; mothers who responded yes to at least one question were the victims of sexual violence [29].

Maternal depression is assessed by the validated Edinburgh postnatal depression tool. Mothers with an Edinburgh postnatal depression score of < 13 were considered to have no depression, and ≥ 13 were considered to have depression [30]. Maternal social support was assessed by the maternity social support scale: low social support < 18, medium social support 19–23, and high social support 24–30 [31]. Husband’s index of pregnancy during the current baby is defined as husband’s intention to have a child before conception [28]. The duration of postpartum period was classified as ≤ 6 weeks and ≥ 7 weeks for the purpose of this study [32]. Dominance in decision-making authority is defined as a unitary form of making decisions on overall household affairs by a husband only, and husband physical fight history is defined as those husbands who have fought with other men or neighbors previously.

The household wealth index is assessed by 38 items containing a household’s ownership of selected assets and properties. Then principal component analysis was done and divided into three equal parts: poor, which was labeled as “1”, medium, labeled as “2”, and rich, labeled as “3” [33]. History of intimate partner violence: Is a condition in which the mother faced at least one of the types of intimate partner violence during or before pregnancy by her husband [34]. The extended postpartum period was defined as the time between the birth of a viable baby and one year [35].

### Data collection instrument and procedures

Face-to-face interviewers administered structured questionnaires used to collect data. The tool was developed from a tool used to assess domestic violence against women in low-income countries and related literature [5, 6, 36]. The tool has six parts: the socio-demographic part; the household wealth assessment tool has 38 items developed from related literature [33], and the reproductive history-related part. In addition, the couples behavior and substance use part has nine questions, and the maternal personal and social-related part has two parts, i.e., the depression tool, which has ten items with a minimum score of zero and a maximum score of three for each item; The depression assessment tool has a minimum score of zero and a maximum score of thirty; and the maternity social support scale has six items with a minimum score of one and a maximum score of five for each item [30].

The Maternity Social Support Scale Tool has a minimum score of six and a maximum score of thirty [31]. Finally, the tool to assess outcome variable and history of intimate partner violence has twenty questions, which were developed from related literature and have an internal consistency of 0.88 [5, 6, 13, 36].

### Data quality assurance

To assure the quality of the data, a pretest was done on 10% of the study participants two weeks before the actual data collection at Birbir town. The content validity and internal consistency of the tool were verified, and Cronbach’s α test of the tool was 0.88. Initially, the tool was developed in the English language and translated to Amharic for actual data collection. Then the Amharic version was retranslated back to English to check the consistency of the tool.

Data collectors were given training for two days on the objectives of the study, data collectors, and participant safety. Following the WHO ethical safety guidelines and recommendation for research on violence against women, four female data collectors and two supervisors were recruited [37]. Supervision was held by supervisors daily to ensure the clarity, accuracy, and consistency of the collected data.

### Data processing and analysis

The data were cleaned, coded, and entered into Epi Data version 3.1 software before being exported to SPSS version 25 software for further analysis. Descriptive statistics such as frequency, percentages, and summary measures were carried out, and the results were presented using narrative form and tables. Both bivariable and multivariable logistic regression analyses were used to identify factors associated with EPIPV. Variables with a p-value < 0.25 in bivariable logistic regression analysis were transferred to multivariable logistic regression to control the effect of confounders.

Both the Crude Odds Ratio (COR) in bivariable logistic regression and the Adjusted Odds Ratio (AOR) in multivariable logistic regression with a 95% CI were calculated to show association and strength of association, respectively. In multivariable logistic regression analysis, variables with a p-value of < 0.05 were declared statistically significant factors. To check the goodness of model fit, the Hosmer-Lemeshow goodness of fit test was used. A multi-collinearity test was carried out to see the correlation between independent variables by using variance inflation factor (VIF) and tolerance (T). Then the final results were presented in tables and narrative form.

## Results

### Socio-demographic characteristics

For the purpose of this study, 570 postpartum mothers were selected, and 555 participated, with a response rate of 97.4%. The participants’ mean age and standard deviation were 28.7 ± 5.8 years, respectively. The minimum age of the study participants was 17 years old, and the maximum was 45 years old. This study revealed 42% of them are housewives, and 87.4% have a formal education. The mean age and standard deviation of the participants’ husband were 34.58 ± 6.053, and 80.2% of them were aged greater than or equal to thirty years. In addition, 50% of their husbands were merchants, and 92.8% had formal education.

Furthermore, 203 (36.6%) of the participants’ marriages were love marriages, in which they got into marriage based on the interests of the couples, and 94.1% of the participants remained with their husband after childbirth. This study found 212 (38.2%) and 187 (33.7%) were at poor and rich economic levels, respectively, while the rest were at medium economic levels (Table 1).

**Table 1:** Socio-demographic characteristics of the study participants (n = 555)

Variables	Categories	Frequency	Percentage
Maternal age	17–23	104	18.80
	24–29	238	42.90
	30–35	134	24.10
	≥ 36	79	14.20
Religion	Orthodox	245	44.10
	Protestant	217	39.10
	Muslim	87	15.70
	Others^*^	6	1.10
Ethnicity	Gamo	271	48.80
	Gofa	97	17.50
	Amhara	104	18.70
	Oromo	58	10.50
	Others**	25	4.50
Maternal Occupation	House wife	233	42.00
	Merchant	129	23.20
	Government employ	117	21.10
	Daily worker	34	6.20
	Student	36	6.50
	Others***	6	1.10
Husband Occupation	Merchant	279	50.30
	Government employ	186	33.50
	Daily worker	53	9.50
	Others***	37	6.70
Maternal educational status	No formal education	70	12.60
	Primary education	209	37.70
	Secondary education	123	22.10
	Diploma and above	153	27.60
Husband educational status	No formal education	40	7.20
	Primary education	171	30.80
	Secondary education	202	36.40
	Diploma and above	142	25.60
With whom the mother lives currently	With husband	522	94.10
	Others****	33	5.90
Who select your current husband	My self	148	26.70
	Love marriage	203	36.60
	Family	91	16.40
	Friends	113	20.30

**Footnote-** *: Catholic; **: Derashe, Gurage, Konso, Silte and Wolayita, ***: non-government work, fisher, farmer; ****: husband family, self-family, her sister, grandmother

### Reproductive characteristics of the study participants

Among the total study participants, 35 (6.8%) were married before the age of 18, and 72.4% were in a relationship for more than four years. In addition, 58% of the study participants were more than or equal to seven weeks after giving birth, and 99.5% of the currently delivered infants were alive. In addition, 53.2% of participants’ husbands planned to have a child during the index of their current baby, and 82% of the study subjects have children less than or equal to three. In addition, 90 (16.2%).

and 12 (2.2%) had a history of abortion and stillbirth before the current baby, respectively, and 5.

(0.9%) of the study participants were HIV-positive mothers (Table 2).

**Table 2:** Reproductive characteristics of the study participants (n = 555)

Variables	Categories	Frequency	Percentage
Age at marriage in year	< 18	35	6.80
	≥ 18	520	93.20
Length of current relationship in year	1–3	153	27.60
	4–6	169	30.50
	7–9	104	18.60
	≥ 10	129	23.30
Sex of current baby	Male	311	56.40
	Female	241	43.60
Duration of postpartum period	0–6 Weeks	233	42.00
	≥ 7 Weeks	322	58.00
Husband’s index of pregnancy during current baby	Yes	294	53.20
	No	258	46.80
Husband’s satisfaction with the gender of current Baby	Yes	388	70.40
	No	164	29.60
Number of alive children	≤ 3	455	82.00
	≥ 4	100	18.00

### Substance use and behavior related factors of the study participants and partners

As identified, 34.8% and 22% of the participant’s husbands are alcohol and Khat users, respectively.

In addition, 13% of participants’ husbands have a history of physical fights with other men and/or.

neighbors, and 18.9% of mothers are alcohol users (Table 3).

**Table 3:** Substance use and behavior of the study participants and their husbands (n = 555)

Variables	Categories	Frequency	Percentage
Maternal alcohol use	Never	450	81.10
	1 to 2 per week	64	11.50
	≤ 3per months	41	7.40
Husbands’ alcohol use	Never	362	65.20
	1 to 2 per week	51	9.20
	≤ 3per months	24	4.30
	Daily	118	21.30
Husbands Khat Chew	Never	433	78.00
	1 to 2 per week	31	5.60
	≤ 3per months	43	7.80
	Daily	48	8.60
Husbands Physical fight history	Yes	72	13.00
	No	483	87.00

### Maternal personal and social related factors

According to this study, 363 (65.4%) of the mother’s household affairs decisions were dominated and decided by their husbands. Of the total study participants, 41.6% had a history of intimate partner violence during or before pregnancy. In addition, 61% of the mothers had low social support during the extended postpartum period, and 20.2% of them suffered from depression (Table 4).

**Table 4:** Maternal personal and social related factors (n = 555)

Variable	Categories	Frequencies	Percentage
Household decision maker	Husband	363	65.40
	Jointly	18	3.20
	Wife	174	31.40
History of intimate partner violence	Yes	231	41.60
	No	324	58.40
Maternal Depression	No depression	443	79.80
	Have depression	112	20.20
Maternal Social support	Low social support	339	61.00
	High social support	216	39.00

### The prevalence of extended postpartum intimate partner violence

The overall prevalence of EPIPV was 45% (95% CI: 40.89, 49.20) in this study setting. This study revealed that sexual violence and control of behavior were the most common specific types of intimate partner violence among extended postpartum mothers, which were 26.7% and 24%, respectively (Table 5).

**Table 5:** Prevalence of extended postpartum intimate partner violence (n = 555)

Types of violence	Categories	Frequencies	Percentages
Physical violence	Yes	99	17.80
	No	456	82.20
Psychological violence	Yes	129	23.20
	No	426	76.80
Economical violence	Yes	96	17.30
	No	459	82.70
Control of behavior	Yes	133	24.00
	No	422	76.00
Sexual violence	Yes	148	26.70
	No	407	73.30
**Overall extended postpartum intimate partner violence**	Yes	250	45.00
	No	305	55.00

### Factors associated with extended postpartum intimate partner violence in Arba Minch town

In this study, bivariable logistic regression analyses showed that husband educational level, husband dominance in decision-making of household affairs, duration of postpartum period, husband’s index of pregnancy during current baby, husband disappointment in gender of current baby, husband alcohol and Khat use, husband physical fight behavior, previous history of intimate partner violence, maternal social support level, and maternal depression were factors with p-values < 0.25 and fitted with a multivariable model.

In multivariable logistic analyses, husband educational level, couple’s alcohol consumption, husband dominance in decision-making, husband disappointment in the gender of the current baby, previous history of intimate partner violence, and maternal social support level were significantly associated factors with EPIPV at p-values < 0.05. This study revealed that the odds of EPIPV among mothers whose husbands have no formal education were 3.62 times higher than those whose husbands achieved a higher level of education (AOR = 3.62; 95% CI: 1.32, 9.91). In addition, the odds of EPIPV among mothers whose husbands have only achieved secondary education were 2.96 times higher than those whose husbands achieved a higher academic level (AOR = 2.96; 95% CI: 1.60, 5.48). The odds of EPPIV were 1.74 times higher among mothers whose husbands use alcohol than whose husbands never use it (AOR = 1.73; 95% CI: 1.06, 2.82).

The odds of EPIPV among mothers whose husbands dominate decision-making authority were 1.94 times higher than those who decide by themselves (AOR = 1.94; 95% CI: 1.13, 3.33). Similarly, the odds of EPIPV among mothers whose husbands were disappointed in the gender of their current baby were 2.13 times higher than their counterparts (AOR = 2.13; 95% CI: 1.28, 3.56), and the odds of EPIPV among mothers who have a previous history of intimate partner violence were 5.71 times higher than those who never experienced it (AOR = 5.71; 95% CI: 3.59, 9.10). Furthermore, the odds of EPIPV among mothers who have low social support were 4.37 times higher than those who have high social support during their postpartum period (AOR = 4.37; 95% CI: 2.53, 7.55) (Table 6).

**Table 6:** Factors associated with extended postpartum intimate partner violence (n = 555)

Variables	Categories	Over all intimate partners violence		COR (95% CI)	AOR (95% CI)
		Yes (250)	No (305)		
Husband educational status	No formal education	30(75%)	10(25%)	9.90(4.39, 22.38)	3.62(1.32, 9.91) *
	Primary	79(46.2%)	92(53.8%)	2.84(1.73, 4.64)	1.20(0.63, 2.29)
	Secondary	108(53.5%)	94(46.5%)	3.80(2.35, 6.12)	2.96(1.60, 5.50) *
	Diploma and above	33(23.2%)	109(76.8%)	1	1
Decision maker of household	Husband	200(55.1%)	163(44.9%)	3.74(2.50, 5.59)	1.94(1.13, 3.33) *
	Jointly	7(38.9%)	11(61.1%)	1.93(0.71, 5.31)	0.74(0.18, 3.00)
	Wife	43(24.7%)	131(75.3%)	1	1
Duration of postpartum period	≤ 6 weeks	116(50.9%)	112(49.1%)	1	1
	≥ 7 weeks	134(41%)	193(59%)	0.67(0.48, 0.94)	0.86(0.54, 1.37)
Husband’s index of pregnancy	Yes	101(34.4%)	193(65.6%)	1	1
	No	146(56.6%)	112(43.4%)	2.49(1.77, 3.52)	1.08(0.67, 1.73)
Husband Satisfaction to gender of current baby	Yes	136(35.1%)	252(]64.9%)	1	1
	No	111(67.7%)	53(32.3%)	3.88(2.63, 5.72)	2.13(1.28, 3.56) *
Husband Alcohol use	Yes	122(63.2%)	71(36.8%)	3.14(2.18, 4.52)	1.73(1.06, 2.82) *
	No	128(35.4%)	234(64.6%)	1	1
Husband Khat use	Yes	67(54.9%)	55(45.1%)	1.66(1.11, 2.49)	1.00(0.59, 1.73)
	No	183(42.3%)	250(57.7%)	1	1
Husband’s physical fight	Yes	43(59.7%)	29(]40.3%)	1.98(1.194, 3.27)	1.04(0.54, 1.99)
	No	207(42.9%)	276(57.1%)	1	1
History of intimate partners violence	No	77(23.8%)	247(76.2%)	1	1
	Yes	173(74.9%)	58(25.1%)	9.57(6.46, 14.16)	5.71(3.59, 9.07) *
Social support	Low	222(65.5%)	117(34.5%)	12.74(8.08, 20.10)	4.37(2.53, 7.55) *
	High	28(13%)	188(87%)	1	1
Maternal Depression	No	167(37.7%)	276(62.3%)	1	1
	Yes	83(74.1%)	29(25.9%)	4.73(2.97, 7.53)	1.32(0.73, 2.38)

**Footnote**: * - indicate factors significantly associated with extended postpartum intimate partner violence at p - value < 0.05 with AOR, AOR – Adjusted odds ratios, COR – Crude odds ratios, “1”- reference group

## Discussion

Postpartum intimate partner violence is a highly prevalent problem with a multidimensional impact on the mother, the infant, family members, and the world as a whole, as it may affect the future generation that takes over the world. Women exposed to intimate partner violence were at higher risk of developing common mental health problems, which might affect infant nurturing [16, 17]; and it might lead to family dissolution, which might increase the number of street children, which indirectly affects the world economy. In addition, it might affect infant immunization status [23]; which increases childhood morbidity and health costs as it affects family members’ economies and maternal psychology.

The prevalence of EPIPV was 45% in this study setting. This was in line with the study conducted in Ghana, which was 46% [15]. Conversely, it was higher than the study conducted in central Ethiopia at 31.4% [28], rural Zambia at 8.1% [14], Australia at 17.4% [11], Nepal at 20% [38] and South Africa at 24.56% [39]. This discrepancy might be due to the difference in the assessment method employed. In addition, studies conducted in central Ethiopia, Australia, Nepal, and South Africa were institution-based study settings that might miss violating mothers and lead to an underestimation of the prevalence of intimate partner violence. Furthermore, the discrepancy between the studies conducted in Zambia might be due to the difference in the study participant’s residence, which might lead to underestimation of this problem as rural residents are less likely to disclose it during data collection.

However, it was lower than the study conducted in Brazil 51.2% [12] and Iran 58% [13]. These discrepancies might be due to differences in socioeconomic status among the study participants. In addition, the difference between the studies conducted in Iran might be due to differences in educational status and means of data collection, i.e., it was a self-administered structured questionnaire that enabled the participants to fill out the questionnaire without any fear since it was a sensitive issue.

The multivariable logistic analysis showed that mothers who have husbands with no formal education and have only a secondary education been more likely to be violated than those whose husbands have achieved a higher level of education. Furthermore, mothers whose husbands dominate in decision-making, mothers whose husbands use alcohol, and mothers whose husbands were disappointed in the gender of the current baby were more likely to be violated than their counterparts. In addition, mothers with a previous history of intimate partner violence and low social support levels were more likely to be violated.

In our study, mothers whose husbands have no formal education and had only secondary education been more likely to be violated than those whose husbands had achieved a higher level of education. This was supported by studies conducted in Hosanna, Ethiopia [40], and the Islamic Republic of Iran [41], respectively. This might be because husbands who have no formal education might have no awareness of the consequences of intimate partner violence, and those who have lower academic achievement have a lower understanding of the consequences of intimate partner violence than those who have achieved a higher level of education. In addition, they might have a lower understanding of the physical and emotional demands of their infant and wife during the postpartum period than those who achieved a higher level of education, as it might provoke intimate partner violence after childbirth.

This study found that mothers whose husbands dominate the overall household decision-making authority were more likely to be violated than those who decide on their own. This was supported by the evidence obtained from the study conducted in central Ethiopia [28]. This might be because husbands who dominate decision-making power are guided by their own perspective alone, which might cause intimate partner violence. In addition, the African proverb says that “He who listens to women suffers from famine at harvest time,“ which might support this evidence as this proverb might encourage husband dominance in decision-making [42], which might aggravate intimate partner violence after childbirth.

Husbands’ alcohol use was also significantly associated with EPIPV. This was supported by the studies conducted in Mumbai [43], Nepal [44], and Malaysia [45]. The possible justification might be that alcohol use might lead to misunderstandings among couples during the postpartum period, and alcohol users might violate their wives during the active phase after use due to the alcohol effect. In addition, husband’s disappointment with the gender of the current baby was significantly associated with EPIPV. This was supported by evidence obtained from the study conducted in Iran [13]. The possible explanation might be due to the husband’s preference for a particular gender, which might lead to EPIPV after childbirth if the gender of their current baby turns out to be different than anticipated.

This study revealed that mothers who had a previous history of intimate partner violence were more likely to be violated than their counterparts. This was corroborated by the studies conducted in Kenya [46], South Africa [39], Nepal [44], and Southern Sweden [47]. The possible reason might be that mothers who were previously violated might be ashamed to disclose their husband’s violent act and remain silent with the hope of change in the future, leading to the continuation of the problem after childbirth.

Similarly, mothers with low social support were more likely to be violated than mothers with high social support. This was supported by the studies conducted in Southern Sweden [47], China [48], and Ghana [15]. The possible explanation might be that during the postpartum period, there might be a significant increase in the physical and emotional demands of family members that might lead to intimate partner violence as she is unable to fulfill them due to an increase in workloads. Furthermore, infant nurturing is a significant burden on postpartum mothers if they do not receive additional support from family members and their husbands during the postpartum period, as it may result in intimate partner violence if she is unable to meet her husband’s expectations during the postpartum period.

### Limitations and strength of the study

As it was a sensitive issue, it might be prone to social desirability bias, and the effect of extended postpartum intimate partner violence was not studied as it was a cross-sectional study. This is the first study that was conducted on postpartum mothers up to one year after childbirth in this study setting.

### Conclusion and recommendation

The prevalence of extended postpartum intimate partner violence was high in this study setting. Therefore, increasing husbands’ educational level, particularly those who have no formal education and lower academic achievement, integrating maternal social support assessment into maternity and child health care services, and incorporating screening of postpartum intimate partner violence into an expanded program for immunization might have paramount value in the prevention and reduction of intimate partner violence after childbirth. In addition, increasing women’s status in the community and increasing the community’s awareness through the media of gender norms might alleviate this devastating problem in the community. Substance users should be identified and counseled by community leaders and religious fathers. Furthermore, screening for intimate partner violence during antenatal care may prevent it after childbirth.

### Electronic supplementary material

Below is the link to the electronic supplementary material.

Supplementary Material 1

## Data Availability

The data used in this study is available upon reasonable request to the corresponding author at any time if required. Therefore, any person who is in need of it can obtain it from the principal investigator through his contact address (e-mail: lemafika2014@gmail.com).

## Declarations

Voluntary written informed consent was obtained before starting the interview. Legally authorized representatives of the minor groups were also obtained. Furthermore, informed consent was taken from mothers under the age of 18 because after they married, they could decide everything about themselves, and due to the nature of the topic, we could not obtain consent from family or husbands [49].

## Abbreviations

EPIPV: Extended Postpartum Intimate Partner Violence
